# Effect of *Pseudomonas protegens* EMM-1 Against *Rhizopus oryzae* in Interactions with Mexican Autochthonous Red Maize

**DOI:** 10.3390/life15040554

**Published:** 2025-03-28

**Authors:** Bruce Manuel Morales-Barron, Violeta Larios-Serrato, Yolanda Elizabeth Morales-García, Verónica Quintero-Hernández, Paulina Estrada-de los Santos, Jesús Muñoz-Rojas

**Affiliations:** 1 Instituto Politécnico Nacional, Escuela Nacional de Ciencias Biológicas, Prolongación Carpio y Plan de Ayala, Col. Santo Tomas, Alcaldía Miguel Hidalgo, Ciudad de México C.P. 11340, Mexico; bio.brucemorales@outlook.com (B.M.M.-B.); viosdatafactory@gmail.com (V.L.-S.); 2 Ecology and Survival of Microorganisms Group, Laboratorio de Ecología Molecular Microbiana, Benemérita Universidad Autónoma de Puebla (BUAP), Edificio IC11, Ciudad Universitaria, Colonia Jardines de San Manuel, Puebla C.P. 72570, Mexico; yolanda.moralesg@correo.buap.mx (Y.E.M.-G.); vquinterohe@secihti.mx (V.Q.-H.); 3 Grupo Inoculantes Microbianos, Facultad de Ciencias Biológicas, Benemérita Universidad Autónoma de Puebla, Puebla C.P. 72570, Mexico; 4 Secretaría de Ciencia, Humanidades, Tecnología e Innovación (SECIHTI), Insurgentes Sur 1582, Col. Crédito Constructor, Alcaldía Benito Juárez, Ciudad de México C.P. 03940, Mexico

**Keywords:** inhibition, antagonism, *Pseudomonas*, phytopathogen, hydroponic system

## Abstract

In the present study, the strain *Rhizopus oryzae* EMM was isolated from germinated autochthonous red maize seeds, which were harvested in a region of San Diego-Buenavista, Papalotla, Tlaxcala, Mexico, where cobs with fungal infections have been observed. This fungal strain caused wilting in the maize seedlings. *Pseudomonas protegens* EMM-1 was tested for its ability to inhibit *R. oryzae* EMM, both in culture media and in association with maize plantlets. *P. protegens* EMM-1 inhibited the growth of *R. oryzae* EMM under all culture media conditions explored. The ability of *P. protegens* EMM-1 to inhibit the growth of *R. oryzae* EMM associated with plants was evaluated in both a hydroponic system and in vermiculite. In both systems, *P. protegens* EMM-1 strongly inhibited the growth of *R. oryzae* EMM. The dry weight of root plants infected with *R. oryzae* EMM and inoculated with *P. protegens* EMM-1 increased to 0.43 g, while that of plants infected only with *R. oryzae* EMM reached just 0.19 g under hydroponic conditions. However, no differences were observed under vermiculite conditions. The dry weight of the aerial region of plants infected with *R. oryzae* EMM and inoculated with *P. protegens* EMM-1 was greater than that of plants infected only with *R. oryzae* EMM, both under hydroponic and vermiculite conditions. These results indicate that *P. protegens* EMM-1 inhibits the infection caused by *R. oryzae* EMM, thereby improving plant growth. Moreover, the genome analysis of *P. protegens* EMM-1 revealed the presence of several genes that potentially encode for antimicrobial compounds, which could strengthen the potential use of *P. protegens* EMM-1 as a biocontrol agent in maize plants.

## 1. Introduction

Autochthonous red maize is a variety cultivated in Mexico. Its importance is cultural, in genetics, and due to nutritional benefits. However, it is produced in low quantities mainly for self-consumption [1]. The production of different maize varieties preserves genetic identity and variability in Mexico [1,2]. The conservation of native maize varieties is essential for food security [3]. Nevertheless, infections with pathogenic microorganisms represent one of the main problems in maize production [4,5,6].

Pathogens affect the growth and development of plants and grains under stored conditions [7], causing significant economic losses and human health issues [8]. In Mexico, several strains of pathogenic fungi have been detected in maize plants, negatively affecting production [9,10]; among them, notable strains include *Fusarium* sp. [11,12] and *Aspergillus* sp. [10,13]. In San Diego-Buenavista, Papalotla, Tlaxcala, Mexico, fungal infections in cobs of autochthonous red maize have been observed, but the fungi have never been isolated, nor have they been associated with plant damage.

*Rhizopus oryzae* is a fungus with the ability to secrete compounds responsible for degrading chemical structures in the corn seed [14,15]; some strains are considered seed endophytes [15,16]. Moreover, several fungi can adapt to fungicides, resulting from the excessive application of these compounds to maize [17].

Beneficial bacteria improve the growth of plants through different mechanisms, such as phytohormone production, nitrogen fixation, phosphorous solubilization, systemic response induction, and direct inhibition [18,19]. The inoculation of bacteria in the rhizosphere, which can inhibit or kill phytopathogenic fungi and compete for space and environmental resources, could be a biocontrol strategy to regulate phytopathogens [20,21,22]. This type of antagonistic mechanism that harbors a bacterium can help determine the compounds it secretes [19,22,23]. *Pseudomonas* is a bacterial genus that can secrete different compounds against fungi, such as lipopeptides [24,25,26] and siderophores [27]. In addition, this bacterium can secrete enzymes, such as chitinases [28,29] and proteases [30,31], which act against the fungal cell wall. The secretion of bacterial compounds may vary depending on environmental conditions [26,32,33]. The stress a bacterium undergoes in competition with other microorganisms can influence the secretion of inhibitory compounds [34].

*P. protegens* is a bacterium that can inhibit the growth of various microorganisms, including some human and plant pathogens, under culture medium conditions [35]. *P. protegens* is adapted to the growth medium where the bacterium thrives [36], and its capability to produce inhibitory compounds can be altered [37]. Bacteria that can produce inhibitory compounds could protect plants against the presence of phytopathogens to increase the production of crops [38,39]; for these reasons, exploring the ability of *P. protegens* EMM1 to protect red maize could be an excellent strategy to increase production.

The objective of this study was to isolate and characterize a fungal strain associated with damage to red autochthonous maize plants after infection and to test the ability of *P. protegens* EMM-1 to inhibit the growth of the isolated fungal strain under both in vitro and in vivo conditions. Some insights into the genome sequence of *P. protegens* EMM-1 related to antagonism are also included.

## 2. Materials and Methods

### 2.1. Bacterial Sources

*Pseudomonas protegens* EMM-1 was isolated from the rhizosphere of red maize and identified by the Ecology and Survival of Microorganisms Group (Benemérita Universidad Autónoma de Puebla, Puebla, Mexico) [40,41]. This bacterium can inhibit various types of bacterial strains [40]. *P. protegens* EMM-1 was stored in 20% glycerol at −70 °C until its experimental use.

### 2.2. Phytopathogenic Fungi Isolation

For the isolation of phytopathogenic fungi, nonsterile autochthonous red maize seeds were germinated on Murashige and Skoog medium at 30 °C for 5 days. This maize variety has been cultivated for a long time in San Diego-Buenavista, Papalotla, Tlaxcala, Mexico [41], and it is designated as Red Maize CRP11-1 TLAX for reference in future studies. After germination, different types of mycelia were observed in the damaged seeds and roots. Mycelial samples were taken with a sterile bacteriological loop and inoculated on potato dextrose agar (PDA) medium for isolation. Several sowings were performed until pure isolates were obtained. For morphological identification, the fungal isolates were incubated in LB and PDA medium for 7 days at 30 °C, after which the macroscopic and microscopic characteristics were observed using an optical microscope [42].

### 2.3. Molecular Identification of Fungi

DNA isolation was performed according to Chávez-Ramírez et al. (2024) [43]. Amplification of the internal regions ITS4 and ITS5 of the ribosomal genes (RNA) 18S-5.8S and 5.8S-28S was performed with the universal primers ITS4 (5-TCCTCCGCTTAT- TGATATGC-3) and ITS5 (5-GGAAGTAAAAG- TCGTAACAAGG-3). The forward and reverse sequences were edited and assembled with ChromasPro (Technelysium Pty Ltd., South Brisbane, QLD, Australia). Nucleotide sequences were analyzed using BlastN against the GenBank nucleotide database (BLAST: Basic Local Alignment Search Tool; National Center for Biotechnology Information (NCBI), National Institutes of Health (NIH) Bethesda, MD, USA). Phylogenetic trees were constructed with the maximum likelihood method using the program PhyML 3.0 under the model GTR + I + G [44]. The reliability of the phylogenetic tree was assessed using bootstrap analysis with 1000 replicates. The resulting phylogenetic tree with bootstrap values was visualized using MEGA 7.0 [45].

### 2.4. Pathogenicity Studies

For the pathogenicity experiments, maize seeds were sterilized and germinated using the methodology described previously [46]. At the same time, the fungal mycelium was incubated for 72 h at 28 °C in liquid LB medium without NaCl and then a suspension of approximately 10^6^–10^8^ CFU/mL mycelium was prepared. Five germinated seeds were placed in the fungus suspension for one hour. Each inoculated germinated seed was grown in the hydroponic system. The hydroponic system consisted of a 400 mL glass bottle, and a copper wire was used to hold the seed to the mouth of the bottle. The copper wire was wrapped around the germinated seed and held on the surface of 100 mL of the Murashige and Skoog medium without sucrose or vitamins (MS modified) [46]. The MS modified medium used in this study contained macronutrients and micronutrients. The added macronutrients (mg/L) were as follows: ammonium nitrate (NH_4_NO_3_)-1650, potassium nitrate (KNO_3_)-1900, monopotassium phosphate (KH_2_PO_4_)-170, magnesium sulfate (MgSO_4_·7H_2_O)-370, and calcium sulfate (CaCl_2_·2H_2_O)-440. The added micronutrients (mg/L) were as follows: manganese sulfate (MnSO_4_·H_2_O)-16.9, zinc sulfate (ZnSO_4_·7H_2_O)-8.6, copper sulfate (CuSO_4_·5H_2_O)-0.025, boric acid (H_3_BO_3_)-6.2, sodium molybdate (Na_2_MoO_4_·2H_2_O)-0.25, potassium iodide (KI)-0.83, and iron-EDTA (FeNaEDTA)-36.7. Additionally, myo-inositol was added at 100 mg/L. A sterile cotton plug was placed as a barrier in the mouth of the bottle to avoid contamination. Five non-inoculated germinated seeds were placed in sterile distilled water for one hour and transferred to the hydroponic system (control treatment). All plants were incubated in a plant chamber for 21 days, with a cycle of 16 h of light/8 h of darkness and 70% relative humidity at 25 °C. After incubation, the damage was described.

### 2.5. Dual-Plate Inhibition Method

The interaction between the antagonistic bacteria and the phytopathogenic fungi was evaluated using the dual-plate method, as described before [47]. Briefly, a 5 mm fungal agar plug from actively growing mycelia was placed in the center of a Luria–Bertani modified medium plate (LBm); this medium did not contain sodium chloride. The plate was incubated for 12 h at 30 °C. Then, 20 µL of a bacterial suspension (approximately 10^8^ cells/mL) was inoculated at three points on the plate at equal distances from each other and 1.5 cm away from the fungal colony. A plate with the fungus was used as control. All of the plates were incubated for 4 days at 30 °C. The antagonistic effect was calculated according to the method of Wang et al. [48] and the experiment was performed with three replicates.

### 2.6. Double-Layer Agar Inhibition Method

In this methodology, *P. protegens* EMM-1 was first grown in LBm broth and incubated at 30 °C for 24 h with reciprocal shaking (180 rpm). Then, a 20 μL drop of bacteria (approximately 10^8^ cells/mL) was placed in the center of an LBm plate and incubated for 48 h at 30 °C. After the incubation, the bacterial colonies were removed with a sterile glass slide, and the plates were exposed to chloroform vapor for 30 min. Then, the plates were left semi-open for 20 min to allow evaporation of residual chloroform. Afterward, the plate was covered with 10 mL of soft LBm agar (8 g/L) inoculated with 50 µL of a fungal suspension containing approximately 10^6^−10^8^ CFU/mL [40]. The antagonistic effect was observed for the presence of an inhibition halo, which was quantified using ImagenJ 1.54g; java 1.8.0_401 (https://imagej.net/ij/ (accessed on 7 January 2025)). The experiment was performed with three replicates.

### 2.7. Simultaneous Inhibition Method

The bacterial and fungal strains were grown in LBm liquid medium, the former for 24 h and the latter for 72 h at 28 °C. Then, 1 mL of the fungal suspension was used for serial dilutions to prepare 50 μL of a fungal suspension (containing approximately 5 × 10^6^ spore/mL); this was streaked on LBm agar plates, and 20 μL of the bacterial strain was placed at the center of the streaked plates. The plates were incubated for 48 h at 30 °C, and the inhibitory halos were evaluated at the end of the incubation period. The experiment was performed in triplicate, and sterile water instead of bacterial suspension was used as a control.

### 2.8. Photographic and Statistical Analysis of Inhibition Plate Assays

The plates were photographed from below, and the distance of the inhibition halo and mycelia growth was analyzed with the digital imaging software ImageJ 1.54g; java 1.8.0_401 (https://imagej.net/ij/ (accessed on 7 January 2025)).

For the dual-plate method, the inhibition percentage was calculated with the following formula:

% I.C.M. = (C − T/C) × 100.

% I.C.M. = The percentage of inhibition of mycelium growth.

I.C.M = Inhibition of mycelium growth

C = The growth of the fungus on the plate.

T = The growth of the fungus on the plate in the presence of bacteria.

For the statistical differences in the inhibition of mycelial growth, the values of the inhibition percentage of mycelial growth were evaluated by analysis of variance (ANOVA). Subsequently, Duncan’s multiple means tests were applied to determine the highest inhibition rate [47].

### 2.9. Microbial Growth and Interaction in Liquid Medium

The effect of *P. protegens* EMM-1 on fungal growth was evaluated in a liquid medium. For this, 3.5 mL of approximately 2 × 10^4^ spores/mL of *R. oryzae* EMM was inoculated in six flasks containing 150 mL of LBm and incubated at 30 °C for 48 h at 40 rpm. After the incubation, 3.35 mL of *P. protegens* EMM-1, approximately 3.1 × 10^8^ CFU/mL, was inoculated in 3 flasks with the fungal growth. As controls, *P. protegens* EMM-1 and *R. oryzae* EMM grew individually in 3 flasks with 150 mL of LBm. All cultures were incubated at 30 °C for 72 h at 40 rpm. During the experiment, the UFC/mL of *P. protegens* was quantified at 0, 24, 48, and 72 using the drop plate method described previously [49]. Briefly, the bacterial cell number was determined by serial dilutions (1:10 *v*/*v*) from each treatment suspension. A 20 µL drop of each dilution was then placed on LB plates and incubated for 24 h. The number of bacteria per milliliter was determined in the dilution where the colonies were countable. The growth of the fungus was estimated at the end of 72 h of incubation. To perform this, the mycelia were separated using an 11 µm pore filter and dried in an oven at 75 °C until a constant dry weight was achieved. Then, the inhibition of mycelium was calculated using the following formula: ((C − T)/C) × 100, where C represents the control measurement and T represents the treatment measurement.

### 2.10. Plant Bioassay Preparation

For this method, *P. protegens* EMM-1 was inoculated in 30 mL of LB liquid medium and incubated for 48 h with reciprocal shaking. The bacterial growth was centrifuged at 4000 rpm for 10 min. Then, the pellet was resuspended in 20 mL of sterile water and centrifuged again. These steps were repeated three times. The cells were divided and resuspended in two 250 mL flasks with 100 mL of sterile water. Finally, the bacterial number was determined using the MSDP method described previously. Briefly, serial dilutions were performed with 100 µL of the bacterial suspension. Then, 1.65 µL was applied on LB plates using a replicator [49]. The suspension ended up with approximately 1 × 10^9^ CFU/mL. For the fungus counting, *R. oryzae* EMM was inoculated in PDA and incubated for 48 h. After the incubation period, 10 mL of sterile water was used to flood the plate, and 5 mL of the suspension with spores was recovered. From the previous suspension, 20 µL was used to quantify the number of spores/mL with a Neubauer chamber. For the mixed microorganism treatment, one flask containing the bacterium was inoculated with spores of *R. oryzae* EMM until a concentration of approximately 1 × 10^3^ spore/mL was reached. To prepare the experiment with the plant, a total of 160 red maize seeds were submerged in 70% ethanol for 10 min, washed with sterile distilled water, and then immersed in 1.5% sodium hypochlorite for 20 min [46]. Then, the maize seeds were washed eight times with sterile distilled water. The seeds were inoculated by submerging them into four liquid suspension treatments: (a) bacteria, (b) fungi, (c) mix of bacteria and fungi, and (d) distilled water for 1 h. The germinated and inoculated seeds were used for evaluation in a hydroponic system, as described below.

### 2.11. Microbial Effect on Maize Plants in a Hydroponic System

A total of 20 germinated and inoculated seeds per treatment were grown in a hydroponic system. The hydroponic system consisted of a 400 mL glass bottle with a copper wire hanging from the bottle mouth to hold the seed. The wire was wrapped around the germinated seed, and it was held on the surface of 100 mL of the Murashige and Skoog medium without sucrose [41]. A sterile cotton plug was placed as a barrier at the mouth of the bottle to avoid contamination. All plants were incubated in a plant chamber with a cycle of 16 h light/8 h darkness and 70% relative humidity at 25 °C. After 10 days, 15 plants were removed and washed with distilled water [50]. The plants were divided into roots and aerial parts; then, the plants were dried in an oven at 75 °C to a constant dry weight, and the data were recorded.

### 2.12. Effect of Microorganisms on Maize Plant Growth in Vermiculite

A total of 20 seeds from each treatment were sown in 50 mL conical tubes with 30 mL of vermiculite. All tubes were placed in a plant chamber with a cycle of 16 h light/8 h darkness at 25 °C for 20 days. After 20 days, the plants were removed and washed with distilled water for the condition in the vermiculite system. The fresh weights of the roots and stems were determined with the help of an analytical balance; thereafter, the plants were dried in an oven at 75 °C to a constant dry weight, and the dry weight data were recorded.

### 2.13. Microbial Adherence and Colonization to the Roots in the Hydroponic and Vermiculite Systems

The microbial adherence to the root system was evaluated 24 h after inoculation [50]. For this, 5 inoculated plants were selected from each treatment, and the roots of each plant were cut and submerged in 5 mL of distilled water and vigorously vortexed for 50 s. Then, 1 mL of suspension was used to determine the bacterial number adhered to the roots using the MSDP method. In this method, 100 µL of the suspension was placed in row A of a multiwell plate. Serial 1:10 dilutions were performed up to row H by transferring 20 µL from the previous well into 180 µL of sterile distilled water using a multichannel pipette. Homogenization was carried out by resuspension for 8 s at each dilution step. The dilutions, along with the initial suspension, were inoculated onto culture media using a sterile replicator, depositing 1.65 µL drops onto Petri plates. Samples were incubated at 30 °C. The number of colony-forming units (CFU/mL) was calculated based on the number of colonies in the countable dilution, adjusting for the inoculation volume and dilution factor. For germinated maize samples, the calculation also included the initial dilution volume to determine the CFU per seed [49]; for this, the spore/mL of *R. oryzae* EMM associated with the root plant was determined using the drop plate method [49]; for this method, a series of 1:10 dilutions were first prepared by transferring 100 µL of the sample into 900 µL of sterile water. Then, using a micropipette, 20 µL from each dilution was placed onto an LB agar plate. The plate was then incubated at 30 °C. After incubation, visible colonies were counted to determine the concentration of viable microorganisms in the original sample. The colonization of microorganisms in the roots of the hydroponic system was quantified at 10 days after inoculation, while in the vermiculite systems, it was quantified at 20 days.

### 2.14. Statistical Analysis for Plants

The data on plants (growth parameters, adherence, and colonization) were subjected to a one-way analysis of variance (ANOVA). Significant differences between averages were obtained with the Duncan multiple range test at *p* < 0.05, using Statical Package for the Social Sciences (SPSS) (Version 28.0.1)

### 2.15. Genome Sequencing

The genomic DNA was isolated using the cetyltrimethylammonium bromide (CTAB) method [51]. The genome sequence was obtained by Novogene (https://www.novogene.com/us-en/ (accessed on 7 January 2025); Novogene Co., Ltd., Beijing, China) using the Illumina NovaSeq 6000 platform (Illumina Inc., San Diego, CA, USA). Annotation was performed using the standard operating procedure at the NCBI Prokaryotic Genome Annotation Pipeline (PGAP) v5.1.

### 2.16. Genome Analysis

The genome was compared in the Type Strain Genome Server (TYGS, https://tygs.dsmz.de/ (accessed on 11 January 2023)) to identify the strain at the species level. Using FastANI v0.1.2 (https://www.kbase.us/ (accessed on 11 January 2023)), the average nucleotide identity was calculated to confirm the strain’s identity. The genome was also analyzed with antiSMASH v7.0 [52] to reveal genetic information for the potential production of antimicrobial compounds.

## 3. Results

### 3.1. Morphological Characterization of Phytopathogens Isolated from Maize Seeds

**Figure 1:**
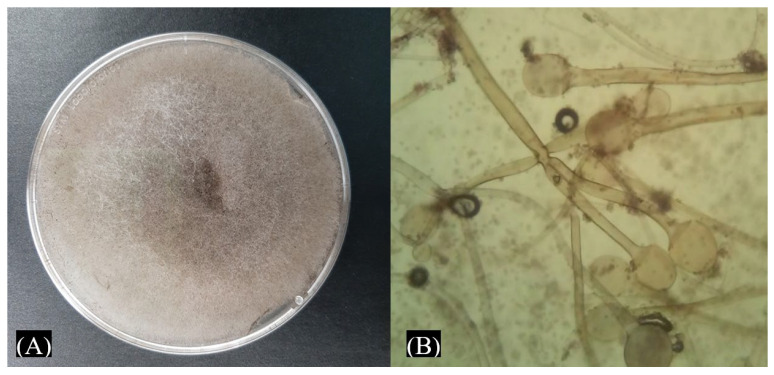
Morphological characteristics of *Rhizopus* (strain EMM) isolated from seeds of autochthonous red maize. (**A**) Colony after 4 days of incubation on PDA. (**B**) Sporangium and sporangiophore.

The maize plants in San Diego-Buenavista, Papalotla, Tlaxcala, Mexico, have been attacked by unidentified fungal strains for a long time. In this study, a fungus that grew abundantly on the affected maize was characterized. This fungus produces mycelium over the germinated seeds and causes damage to the roots. The fungus shows erect and brown sporangiophores with grouped rhizoids, and the sporangiophores are ellipsoidal-spherical and present a large form with an ellipsoidal or spherical apophysis at the end of the sporangiophores (Figure 1). Sporangia are globular and contain a columella at the base; the colony covered the PDA medium in 24 h and produced spores. The fungus characterization was compared with the description of Kortekamp (45), and it was inferred as a possible *Rhizopus* sp. This fungal strain was called EMM.

### 3.2. Molecular Identification of Fungal Strains

**Figure 2:**
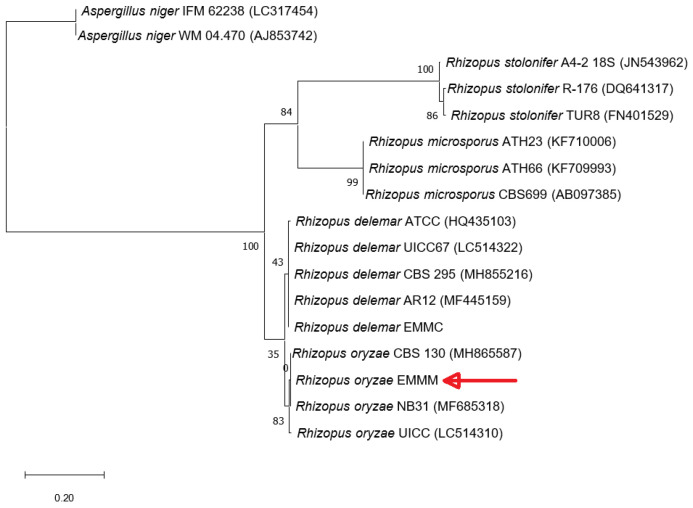
Phylogenetic analysis of *Rhizopus* species, comparing the internal regions ITS4 and ITS5 of the ribosomal genes (RNA) 18S-5.8S and 5.8S-28S using the maximum likelihood method. The bar indicates the number of changes per site, and the numbers in the tree indicate the probability between strains. The red arrow indicates the strain studied. The accession numbers are included in parentheses. *Aspergillus niger* was used as an outgroup.

An analysis of the amplified intergenic region of the isolated EMM showed 621 bp (GenBank accession number: ON365804), and alignment with BlastN showed 100% identity with *R. oryzae* (heterotypic synonym *Rhizopus arrhizus*). The phylogenetic analysis with different species of the genus *Rhizopus* grouped the strain EMM into the group of *R. oryzae* (Figure 2).

### 3.3. Pathogenicity Assays

**Figure 3:**
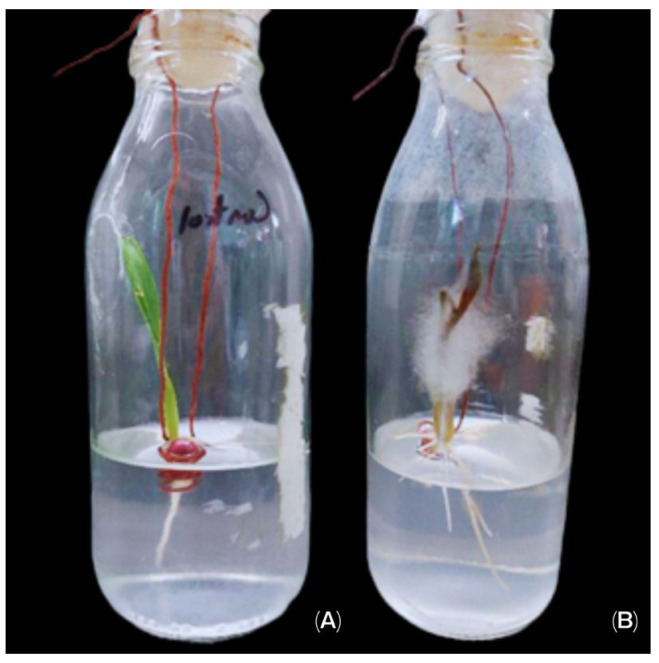
The effect of fungi on plants compared to control. (**A**) Control treatment of autochthonous red maize. (**B**) Maize inoculated with *R. oryzae* EMM.

Maize plants inoculated with *R. oryzae* EMM showed symptoms of disease and mycelium invasion. The *R. oryzae* EMM mainly invaded the roots and caused root rot in all plants, and the plants showed wilting in the first five days (Figure 3). In the control treatment, no lesions were observed.

### 3.4. The Effect of P. protegens EMM-1 Against R. oryzae EMM Under In Vitro Conditions

**Figure 4:**
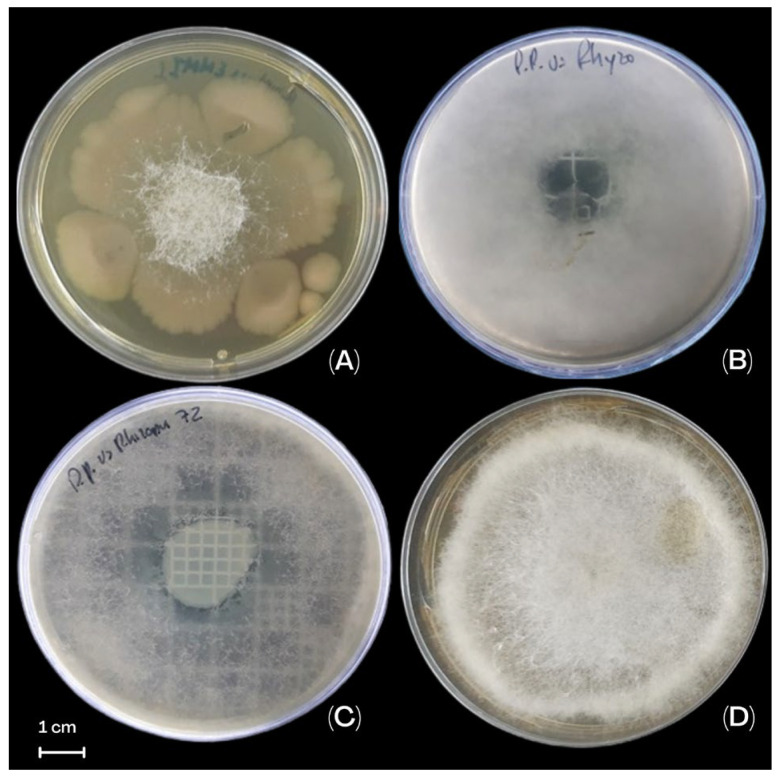
Effect of the *P. protegens* EMM-1 against the *R. oryzae* EMM. (**A**) Interaction in the dual-plate method. (**B**) Interaction in the double-layer method. (**C**) Interaction in the simultaneous method. (**D**) Control of *R. oryzae* EMM.

The dual-plate inhibition analysis showed the activity of *P. protegens* EMM-1 against *R. oryzae* EMM with a statistical difference compared to the control (*p* < 0.05) (Figure 4). The inhibition of the fungus increased over time, showing that at 96 h, the inhibition was 83.1 ± 3.6% (Table 1). In the double-layer inhibition analysis, the effect of *P. protegens* EMM-1 in the growth of *R. oryzae* EMM was observed with the manifestations of clear zones of inhibition starting at 24 h and becoming clearer at 48 h (Figure 4, Table 1).

The simultaneous inhibition analysis revealed that at 48 h, *R. oryzae* completely covered the Petri dish used as the control. When the fungus was grown simultaneously with the bacterium *P. protegens* EMM-1, it inhibited the mycelia of *R. oryzae* EMM (Figure 4, Table 1).

### 3.5. Microbial Interaction in Liquid Medium

**Figure 5:**
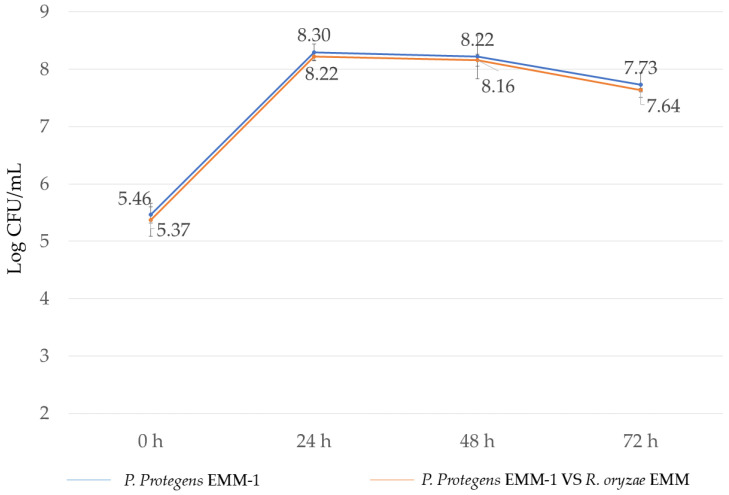
Growth of *Pseudomonas protegens* EMM-1 alone or in interaction with *Rhizopus oryzae* EMM in liquid medium.

The effect of *P. protegens* EMM-1 on the growth of *R. oryzae* EMM in a liquid medium resulted in mycelial inhibition compared to the control without bacteria. The inhibition was quantified by comparing the weight of the mycelium in the control with that in the presence of *P. protegens* EMM-1. The observed inhibition was 84.37%. There were no differences in the growth of the bacteria individually or with the fungus (Figure 5).

### 3.6. Microbial Effect on Maize Plant Growth in a Hydroponic System

**Figure 6:**
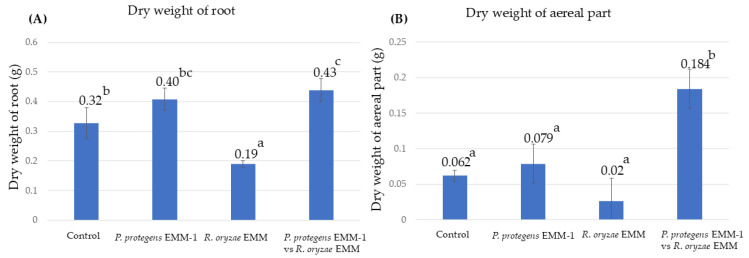
Microbial effect on red maize growth in a hydroponic system. (**A**) Dry weight of roots inoculated with four treatments. (**B**) Dry weight of stem inoculated with four treatments. Data are presented as the mean of five plants per treatment in a hydroponic system. Identical letters within the treatments in the graph indicate that they do not differ significantly at *p* < 0.05.

The capacity of *P. protegens* EMM-1 to inhibit the phytopathogen *R*. *oryzae* EMM was evaluated in an in vivo system, where the dry weight of the aerial region and the root system was obtained. In this experiment, it was observed that the phytopathogen reduced the root dry weight, showing statistically significant differences compared to the control treatment, the treatment where the bacterium was inoculated alone, and the treatment where mixed microorganisms were applied to the plant (Figure 6A). Regarding the aerial dry weight, the combination of the two microorganisms showed a significant increase compared to the application of each microorganism individually or to non-inoculated plants (Figure 6B).

### 3.7. Microbial Adherence and Colonization to the Roots in the Hydroponic System

**Figure 7:**
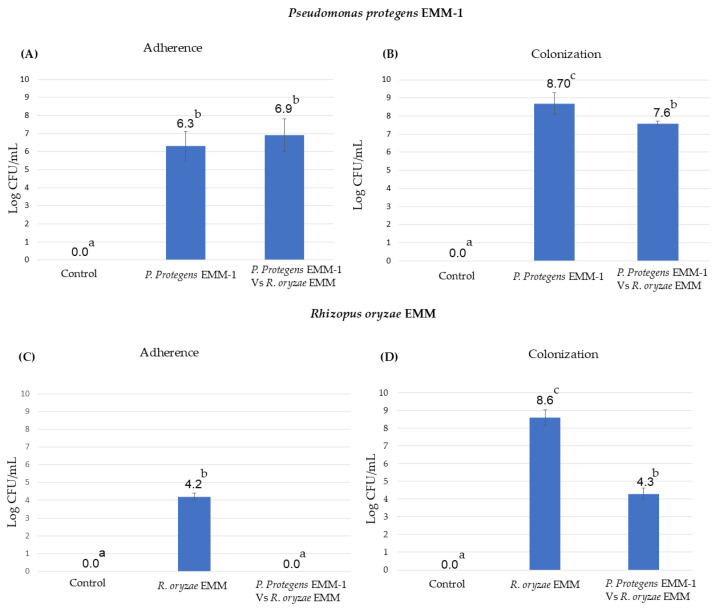
Effect of adherence and colonization of *P. protegens* EMM-1 and *R. oryzae* EMM in roots of maize plants. (**A**) Adherence of *P. protegens* EMM-1 in four treatments. (**B**) Colonization of *P. protegens* EMM-1 at 10 dpi in maize seedlings. (**C**) Adherence of *R. oryzae* EMM in four treatments. (**D**) Colonization of *R. oryzae* EMM at 10 dpi in maize seedlings. Identical letters within the treatments in the graph indicate that they do not differ significantly at *p* < 0.05. The data represent the mean of five determinations.

In this experiment, it was observed that *P. protegens* EMM-1 can adhere to (24 h dpi) and colonize (10 dpi) the roots of red maize (Figure 7A,B). The adherence of *P. protegens* EMM-1 in plants inoculated with the bacterium alone or in combination with *R. oryzae* EMM was in the order of 10^6^ CFU/mL, and the colonization was approximately in the order of 10^7^ CFU/mL. On the other hand, *R. oryzae* EMM was not detected to be adhering to the plant roots at 24 h (Figure 7C). However, at 10 dpi, the fungus heavily colonized the plant roots (fungus-only treatment), but when it was in contact with the bacterium, the fungus decreased the colonization, showing statistical differences with the fungus inoculation treatment (Figure 7D).

### 3.8. Microbial Effect on Maize Plants in a Vermiculite System

**Figure 8:**
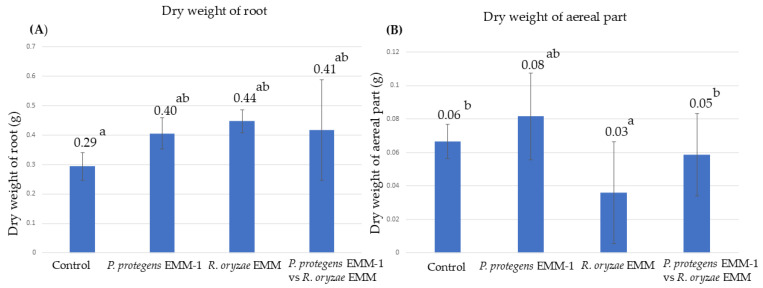
Microbial effect on red maize growth in a vermiculite system. (**A**) Dry weight of roots inoculated with four treatments. (**B**) Dry weight of stem inoculated with four treatments. Data are presented as the mean of 15 plants in vermiculite system. Identical letters within the treatments in the graph indicate that they do not differ significantly at *p* < 0.05.

A vermiculite system was chosen to evaluate antagonistic capacity in a soil-like substrate. After 20 days, the plants were removed from the vermiculite system. In this system, the dry weight of roots increased for treatments inoculated with either bacteria or fungus compared to non-inoculated control plants, while the co-inoculation treatment showed no differences compared to the control or the single inoculations. However, the plants inoculated only with *R. oryzae* EMM showed a low aerial dry weight compared to the control treatments and exhibited symptoms of infection. However, the plants co-inoculated with *P. protegens* EMM-1 and *R. oryzae* EMM showed an improved aerial dry weight, similar to when the pathogenic fungus was not present (Figure 8). It is important to note that the plants in this system did not die from *R. oryzae* EMM infection, unlike those in the hydroponic system.

### 3.9. Microbial Adherence and Colonization to the Roots in the Vermiculite System

**Figure 9:**
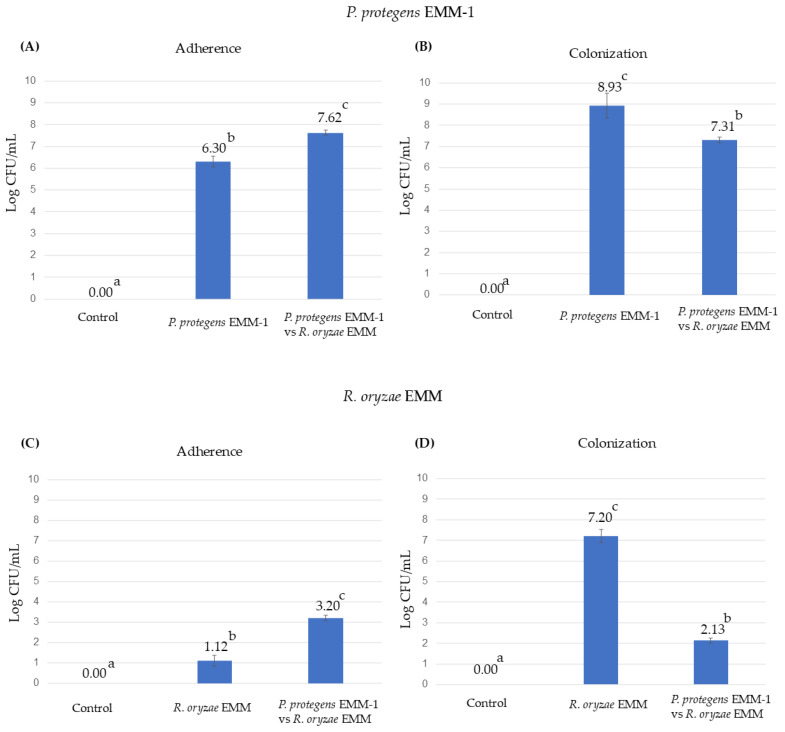
Effect of adherence and colonization of *P. protegens* EMM-1 and *R. oryzae* EMM in roots of maize plants. (**A**) Adherence of *P. protegens* EMM-1 in four treatments. (**B**) Colonization of *P. protegens* EMM-1 at 20 dpi in maize seedlings. (**C**) Adherence of *R. oryzae* EMM in four treatments. (**D**) Colonization of *R. oryzae* EMM at 20 dpi in maize seedlings. Identical letters within the treatments in the graph indicate that they do not differ significantly at *p* < 0.05. The data represent the mean of five determinations.

Under these conditions, the adherence and colonization of *P. protegens* EMM-1 were evaluated (Figure 9). The adherence of *P. protegens* EMM-1 in the mixed treatment increased compared to the treatment with only *P. protegens* EMM-1. However, the opposite occurred during the colonization process, where the treatment with only *P. protegens* EMM-1 showed higher colonization than the mixed treatment (Figure 9). The adherence of *R. oryzae* EMM also increased when interacting with *P. protegens* EMM-1. However, the ability of *R. oryzae* EMM to colonize the plant rhizosphere was significantly inhibited in interaction with *P. protegens* EMM-1 compared to the treatment with *R. oryzae* EMM alone. The effect of the mixed microorganism treatment suggests that *P. protegens* EMM-1 exerts a strong inhibitory effect against *R. oryzae* EMM, protecting the plants from infection and reducing the fungal population.

### 3.10. Genome Analysis Results

The draft genome sequence contained 116 scaffolds (N50 104,758) with 6,976,776 bases and a GC content of 63.4%. A total of 6517 genes were obtained, with 6442 protein-coding genes, 75 rRNA, and 66 tRNA. The result in the TYGS showed that the strain EEM-1 was identified as *Pseudomonas protegens*, with 90.7% similarity to *P. protegens* CHA0^T^, establishing 70% as the value to define a species [53]. The ANI results showed that strain EMM-1 is 98.8% similar to *P. protegens* CHA0^T^, with 95–96% being the value to define a species.

### 3.11. AntiSMASH Genome Analysis

The results from antiSMASH 4.0 showed seven secondary metabolite gene clusters in the genome of *P. protegens* EMM-1 (Table 2), including gene clusters of pyoverdine, orfamide A/orfamide C, 2,4-diacetylphloroglucinol, pyrrolnitrin, enantio-pyochelin, APE vf, and pyoleuteorin.

### 3.12. Data Availability

The draft genome sequence of strain EMM-1 was deposited (23 May 2023) in NCBI GenBank under the accession number JASKHY010000000 with BioProject and BioSample accession numbers of PRJNA974887 and SAMN35301204, respectively. The SRA accession number is SRR24843790. The genome was also deposited (7 June 2023) at the Joint Genome Institute, Walnut Creek, CA, USA, with the numbers Study ID: Gs0161852 and Project ID: Gp0744233.

## 4. Discussion

Mexico produces more than half of the 18 million tons of maize consumed in the world [54]. However, more than 95% of the maize produced in Mexico is white, leading to a loss of genetic variability [54,55]. The red maize belongs to varieties less cultivated by farmers [4,56]. However, it is important because it is considered a functional food due to its high antioxidant content [57,58]. Nevertheless, crop production could be affected by the attack of phytopathogenic fungus [5,59,60] at different stages of plant growth and under stored conditions of seeds diminishing crop quality. In this study, a fungus responsible for damaging germinated seeds of native red maize and causing injury at the seedling stage was isolated. The fungus was characterized by using optical microscopy methods and sequencing of the ITS region [43], revealing that this strain is closely related to *R. oryzae*. Strains meeting these criteria have been classified as members of this species [61].

Identifying the presence of pathogenic fungal strains in autochthonous maize seeds is essential for developing targeted strategies to protect the seeds from mycelial proliferation. Different fungal genera have been isolated from maize seeds, and these genera are responsible for several damages in seed germination [62]. For example, strains of *Aspergillus* sp. and *Fusarium* sp. were isolated from maize seeds disinfected with 1% sodium hypochlorite [63]. *R. oryzae* has been described as a phytopathogen in other crops [42,64,65,66] and has an incidence of 70–80% in stored maize seeds [15,59,67]. To date, *Rhizopus oryzae* has not been reported as a phytopathogen of maize plantlets. However, its presence in seeds and its role as a contaminant of maize seeds have been documented during storage [59,67,68]. In the case of Papalotla, Tlaxcala, autochthonous red maize cobs are affected before harvest, and although “healthy” maize ears are selected for replanting, many of the new seeds experience germination damage. In the present work, *R. oryzae* EMM was isolated from germinated autochthonous maize red seeds and found to damage red corn seedlings, which highlights that this fungus acts as a pathogenic agent under the explored conditions.

The use of inoculants that exert a biocontrol effect is an alternative for inhibiting the infection of pathogenic strains in plants [39,60]. Furthermore, fungicide resistance in fungal strains leads to losses in the production of various crops [69]. Bacteria with the ability to inhibit the growth of fungi have the potential to be used as biocontrol agents for plant diseases [26,70]. Some of these bacteria can secrete different compounds, such as enzymes [71] and secondary metabolites [23]. The method to evaluate the inhibition of fungal strains by bacteria could show an antagonistic effect against fungi; for example, if the evaluated bacterial strain presents an inhibition halo, the secretion of some secondary metabolites like siderophores [23,72] or lipopeptides [24] could be inferred. However, if bacterial overgrowth is observed to degrade mycelium growth, then the compounds involved in the inhibition could be produced by enzymes [71]. In the present work, *P. protegens* EMM-1 was able to inhibit the growth of *R. oryzae* EMM, showing an inhibitory halo with the agar double-layer method and simultaneous inhibition method. The effectiveness could be increased when the bacterial strain was present 24 h before the fungi, in comparison to simultaneous growth, or if the bacterium was inoculated after the mycelium growth [26,73].

The antagonistic capability of *P. protegens* EMM-1 has also been described against *Aspergillus* sp., *Botrytis* sp., *Fusarium* sp., and *Rhizopus* sp. [40]. Secreted compounds such as siderophores and HCN are involved in the fungal inhibition and produce an inhibitory halo [74,75]; both compounds are secreted by *P. protegens* [76,77]. We inferred that the presence of the fungus influences the secretion of inhibitory compounds. In the dual-plate method, when the fungal strains are inoculated before the bacteria in the medium, *P. protegens* EMM-1 surrounds and grows on the mycelium, inhibiting it; this type of antagonism in the dual-plate method was reported in *B. licheniformis* LG against *Rhizopus* sp. [47]. The change in the antagonistic effect could be induced by the presence of the fungus, and the secretion of *P. protegens* EMM-1 could change to inhibit the competitor in the culture medium. The activation of *quorum sensing* in the presence of competitors and subsequent activation of the production of antifungal compounds have been reported for *P. fluorescens* [78]; this process is activated for a better capability to acquire nutrients in the medium [79]. In the environment, bacteria have to fight and survive interacting with other microorganisms, and they develop different strategies to inhibit competitors [23,77]. The interaction evaluated under the simultaneous inhibition method shows what happens when two microorganisms grow and adapt at the same time in the medium [23,80]. Under the antagonism experiments of the dual-plate method, the explored bacteria could secrete enzymes to inhibit the growth of the fungal strain, thus preventing overgrowth. *Pseudomonas protegens* can secret enzymes against *Saprolegia* spp. [81]. In addition, *P. protegens* also can secrete proteases, lipases, and chitinases to inhibit *B. cinerea*, *A. niger*, *Mucor* sp., and *A. flavus* by more than 60% [76]. *Pseudomonas protegens* EMM-1 do not always have the same action; it is possible that *P. protegens* EMM-1 change the secretion of compounds depending on the way it interacts with its competitor.

Evaluating the antagonistic effect under varying conditions is crucial. To demonstrate this, we conducted experiments in a liquid medium, which allowed us to confirm the inhibition against *R. oryzae* EMM by *P. protegens* EMM-1 under this condition. Some fungi produce compounds that can inhibit bacterial growth, which affects the bacteria’s ability to protect plants or adapt to the root environment [82]. However, *R. oryzae* EMM does not produce substances that affect the growth of *P. protegens* EMM-1 because this bacterium was not affected during interaction with the fungus. This is significant for its potential application in hydroponic systems, and we can infer that the inhibitory substances are continuously produced [83]. Orfamide lipopeptides have been identified as potential substances constantly secreted by *Pseudomonas* against phytopathogenic fungi [35].

The germination of autochthonous red maize seeds can be affected by the presence of *R. oryzae* EMM; identifying and studying their pathogenicity will benefit the conservation of these maize varieties [15,39]. In hydroponic systems, *R. oryzae* EMM-1 does not cause damage to the root in the presence of the *P. protegens* EMM-1 under different treatments. Treatment with the interaction of *P. protegens* EMM-1 and *R. oryzae* EMM increased the growth of plants compared to other treatments, and the same effect was observed in the vermiculite system.

We observed that the maize interacting with both microorganisms (fungus and bacterium) grew to the same size as that inoculated with only *P. protegens* EMM-1, demonstrating that it protects maize from fungal infection. This is in accordance with the fact that other strains of *P. protegens* can protect plants and produce hormones that enhance plant growth [37,84,85].

It is currently known that the different compounds present in root exudates are involved in the antifungal activity of many bacterial species [86]. Due to the versatility that *P. protegens* EMM-1 presents to inhibit *R. oryzae* EMM, its action was evaluated under co-interaction with red maize. *P. protegens* EMM-1 exhibits the ability to adhere and colonize the rhizosphere, like other rhizospheric bacteria [41,50], and to inhibit the growth of *R. oryzae* EMM. The plants inoculated with *P. protegens* EMM-1 showed statistically significantly greater biomass than the plants inoculated with *R. oryzae* EMM. In fact, the root dry weight was higher for inoculated plants. Therefore, the capability as a plant growth-promoting bacteria of *P. protegens* EMM-1 could be stimulated during the co-interaction with *R. oryzae* EMM, as shown in other interaction models [87].

Moisture promotes fungal growth [88]. Therefore, in a hydroponic system, ideal conditions were established for *R. oryzae* EMM to proliferate. Under vermiculite conditions—a lightweight, porous mineral material known for its high water and nutrient retention capacity—these conditions could also promote fungal growth. However, in this study, *P. protegens* EMM-1 inhibited fungal growth in both conditions, demonstrating its inhibitory capacity even in environments favorable for fungal proliferation. Future studies should evaluate other conditions, such as soils with varying humidity and temperature, to confirm whether the bacterial strain can consistently inhibit fungal growth. However, under different conditions, the ability of various strains of *Pseudomonas protegens* to inhibit pathogens such as *Botrytis cinerea*, *Phytophthora nicotianae*, *Alternaria alternata*, and *Xanthomonas citri* has been reported in association with plants, highlighting the versatility of this species in exerting antagonism against pathogens across different scenarios [89,90]. Long-term experiments are also necessary to assess whether continuous protection is provided or if bacterial reinoculation is required for the effective biocontrol of phytopathogenic fungi [91].

In this work, *Pseudomonas protegens* EMM-1 was shown to demonstrate a remarkable ability to colonize and proliferate within the rhizosphere of red maize (*Zea mays*) in both systems evaluated. This is attributed to the bacterium’s strong adhesion mechanisms, which enable it to effectively adhere to the root surfaces [92,93]. The adhesion of *P. protegens* EMM-1 to the maize roots could be facilitated by specific cell surface proteins and extracellular polysaccharides, which promote biofilm formation, as reported in other beneficial strains [92]. Some studies have shown that bacterial adhesion and colonization in a plant system demonstrate the potential of these bacteria to be used as bacterial inoculants [20].

The evidence of its action in hydroponic systems and vermiculite demonstrates that *P. protegens* EMM-1 is an excellent candidate for application in soils, where it can establish itself and exert an inhibitory effect. Studies have shown that *P. protegens* E1BL2 is effective when applied in bacterial consortium, enhancing biocontrol and promoting the growth of maize varieties, and this bacterium was isolated from Jala Maize [94].

The application of *P. protegens* EMM-1 to different maize varieties is an opportunity for the future, which could contribute to the health of maize plants and the preservation of maize diversity. In Mexico, conserving the diverse maize species is essential. Therefore, identifying methods to enhance their production is key to ensuring their preservation. Using inoculants with bacteria isolated from various maize varieties is a promising approach, as it suggests that their adaptability to soil and interaction with microbiomes can foster positive development [94,95,96,97].

It is important to identify the types of inhibitory substances encoded in the genome of *P. protegens* EMM-1. For this, we sequenced the genome and analyzed the antiSMASH genome, which shows clusters of secondary metabolites with the capacity to inhibit fungus. This includes 2,4-diacetylphloroglucinol, a metabolite previously reported in the secretion of *P. protegens* [89,98], and orfamide A/orfamide C, which could be responsible for the inhibition of *R. oryzae* EMM-1 [35,99,100]. These compounds have been identified as key factors when a *P. protegens* strain exerts biocontrol effects against fungi. It has been reported that orfamide A affects hyphal development and fungal sporulation [35]. In other studies, 2,4-diacetylphloroglucinol has also been reported to affect the development of infections in plants and has shown effects in plate assays [89,98,101,102].

However, further research is needed to link the detected genes with the inhibition observed in plants. The identification of different clusters of secondary metabolites and the variation in the in vitro assays suggest that *P. protegens* EMM-1 expresses different metabolites simultaneously, which could be the key to its ability to inhibit *R. oryzae* EMM-1 in vitro assays or when interacting with maize.

Our study demonstrates that seed inoculation with the *P. protegens* EMM-1 has an antagonistic effect on the proliferation of *R. oryzae* EMM-1. Additionally, we confirmed the colonization ability of *P. protegens* EMM-1, suggesting that this colonization may persist over time. However, we believe that a foliar application of the bacterium at a later stage of maize development could help sustain its antagonistic effect throughout all growth stages [20]. We believe that our experimental model presents a promising approach to benefit Mexican maize varieties, highlighting the characteristics of *P. protegens* EMM-1 to induce biocontrol effects and enhance autochthonous red maize growth. This will be tested in agricultural production soils to support the conservation of these varieties.

## 5. Conclusions

The germination of autochthonous red maize is susceptible to fungal attack, and the attacking fungi can kill the plantlets or limit their development. For this reason, the control of pathogenic fungi, such as *R. oryzae* EMM (heterotypic synonym *Rhizopus arrhizus*), is necessary to improve the quality of stored grain and grain production.

The *P. protegens* strain EMM-1 exhibits significant potential as a biocontrol agent against *R. oryzae* EMM. In vitro studies have demonstrated its capability to inhibit mycelial growth by up to 80%. Furthermore, the presence of *R. oryzae* does not adversely affect the growth of *P. protegens* EMM-1, underscoring its efficacy as a biocontrol agent. *P. protegens* EMM-1 effectively reduced infection in red maize cultivated in both hydroponic and vermiculite systems.

This bacterium has eight metabolism biosynthetic gene clusters that can produce compounds with an antagonist effect. Therefore, *P. protegens* EMM-1 can benefit the growth of red maize and protect the plant from fungal attack.

## Figures and Tables

**Table 1:** The effect of *Pseudomonas protegens* EMM-1 against the mycelium of *Rhizopus oryzae* EMM was quantified as a percentage, and its impact was assessed using the dual-plate inhibition method and the evaluation using the double layer and simultaneous inhibition methods is expressed in inhibition of halo (mm).

	*R. Oryzae* EMM Inhibition (%)			
	24 h	48 h	72 h	96 h
*P. protegens* EMM-1	11.5 ^a^	64.3 ^c^	72.1 ^d^	83.1 ^d^
	Double layer		Simultaneous inhibition	
*P. protegens* EMM-1	1.9 ^a^ mm		9.0 ^b^ mm	

The letters mean *p* < 0.05. Identical letters within the treatments in each line indicate that they do not differ significantly at *p* < 0.05.

**Table 2:** Potential antimicrobial compounds identified in the genome of *Pseudomonas protegens* EMM-1.

Region	Type	From	To	Most Similar Known Cluster	Similarity (%)
1.1	NRP-Metallophore	1	79,402	Pf-5 pyoverdine	40
18.1	NRPS	127,730	202,218	orfamide A	94
21.1	T3PKS	111,433	147,971	2,4-diacetylpoloroglucinol	100
31.1	Other	1	40,770	pyrrolnitrin	100
31.2	NRPS	129,658	156,887	enantio-pyochelin	60
77.1	Arylpolyene	1	38,410	APE Vf	40
102.1	T1PKS	5341	10,638	pyoluteorin	100
104.1	NRPS	53,410	105,438	Pf-5 pyoverdine	21

Nonribosomal peptide synthetase (NRPS); polyketide synthase (PKS).

## Data Availability

Data are contained within the article.
